# Upper Lip Reconstruction With the Anchor Flap: A Modified Technique of the Reverse Yu's Flap

**DOI:** 10.7759/cureus.80405

**Published:** 2025-03-11

**Authors:** Masashi Kimura, Kotaro Adachi, Tatsuya Kataoka, Shoki Fujita, Shintaro Suzuki

**Affiliations:** 1 Department of Oral and Maxillofacial Surgery, Toyokawa City Hospital, Toyokawa, JPN; 2 Department of Oral and Maxillofacial Surgery, School of Dentistry, Aichi Gakuin University, Nagoya, JPN

**Keywords:** advancement flap, anchor, local flap, rotation flap, vermilion

## Abstract

Various reconstruction methods using local flaps have been reported for upper lip defects. The reverse Yu's flap is a reconstruction method consisting of three flaps including the advancement flap, rotating flap, and mucosal flap. It offers the advantage of low risk of microstomia occurrence because this method restores the form of the vermilion using buccal mucosa as a mucosal flap. Herein, we report the case of a 77-year-old Japanese man who underwent reconstruction using a modified reverse Yu's flap for a defect following the resection of right upper lip basal cell carcinoma. The patient presented with a 4.2×3.5 cm mass on the right upper lip. Resection was planned, and the anticipated defect in the vermilion following resection measured approximately 3.5 cm, making primary closure unfeasible. Thus, the defect was reconstructed using a reversed Yu's flap with minor modifications to accommodate the larger defect. The modifications to the technique are described herein and have been designated as the "anchor flap" due to the characteristic shape of the incision line.

## Introduction

Various reconstruction methods have been reported for lip defects using local flaps or free-flap reconstruction, depending on the location and size of the defect [1-5]. While both techniques have advantages and disadvantages, local flaps are preferred due to their superior color and texture matching, as well as their similarity to the surrounding skin. Furthermore, this method does not require additional surgery for the donor site, and it allows earlier patient recovery. Therefore, local flaps should be the first choice for medium-sized defects [1,2]. However, due to the relatively lower incidence of upper lip cancer compared to lower lip cancer, reported reconstructive techniques for upper lip defects are limited [2]. Yu reported a reconstruction method for lower lip defects using a local flap combining rotating and advancement flaps [3]. This technique can be applied to the reconstruction of an upper lip defect and is referred to as the reverse Yu's flap [1,2,6,7]. In this report, we describe a modified version of this method to address larger defects, which we have termed the "anchor flap" due to the characteristic shape of its incision line.

## Case presentation

**Figure 1:**
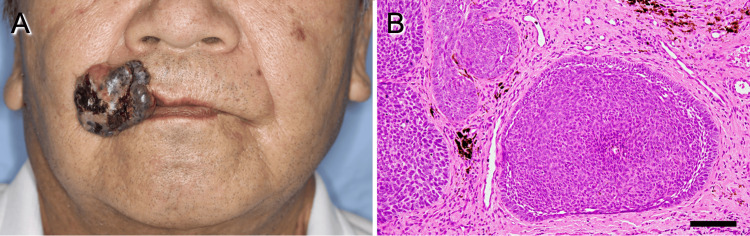
Preoperative view of the patient. (A) Clinical examination showed a mass measuring 4.2×3.5 cm mass on the right upper lip. (B) Histopathology of the biopsy specimen showed basal cell carcinoma (hematoxylin and eosin stain), ×200 magnification (scale bar: 100 μm).

A 77-year-old Japanese man presented to our hospital with a 4.2×3.5 cm painless, easily hemorrhagic mass on the right upper lip (Figure 1A). His medical history included hypertension and thymic cyst enucleation. An incisional biopsy confirmed a histological diagnosis of basal cell carcinoma (Figure 1B).

**Figure 2:**
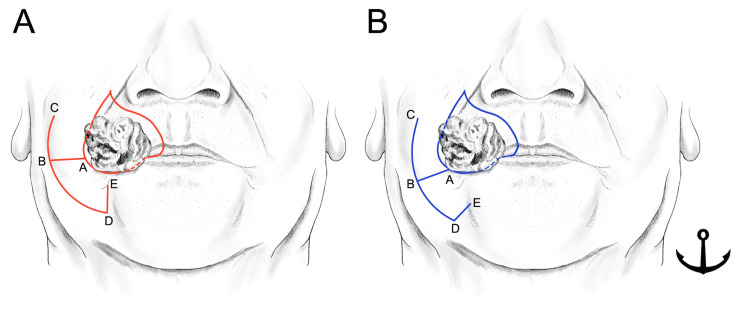
Incision lines of the reverse Yu's flap and modified reverse Yu's flap (anchor flap). (A) Incision lines of the reverse Yu's flap. (B) Incision lines of the modified reverse Yu's flap (anchor flap). A horizontal incision to create the rotating flap running laterally from the corner of the mouth (A-B) was modified to fit the form of the vermilion by tilting it slightly downward. Besides, designing the rotating flap (B-D-E), the original method showed that a vertical line (D-E) is designed as perpendicular to the horizontal incision (A-B) from the arcuate line, and its length should be half the distance of the D-A. The authors designed this vertical line to tilt slightly toward the midline to maintain the blood supply of the rotating flap (B, D-E). Image Credits: Figure 2 is an original image created by the author Masashi Kimura.

**Figure 3:**
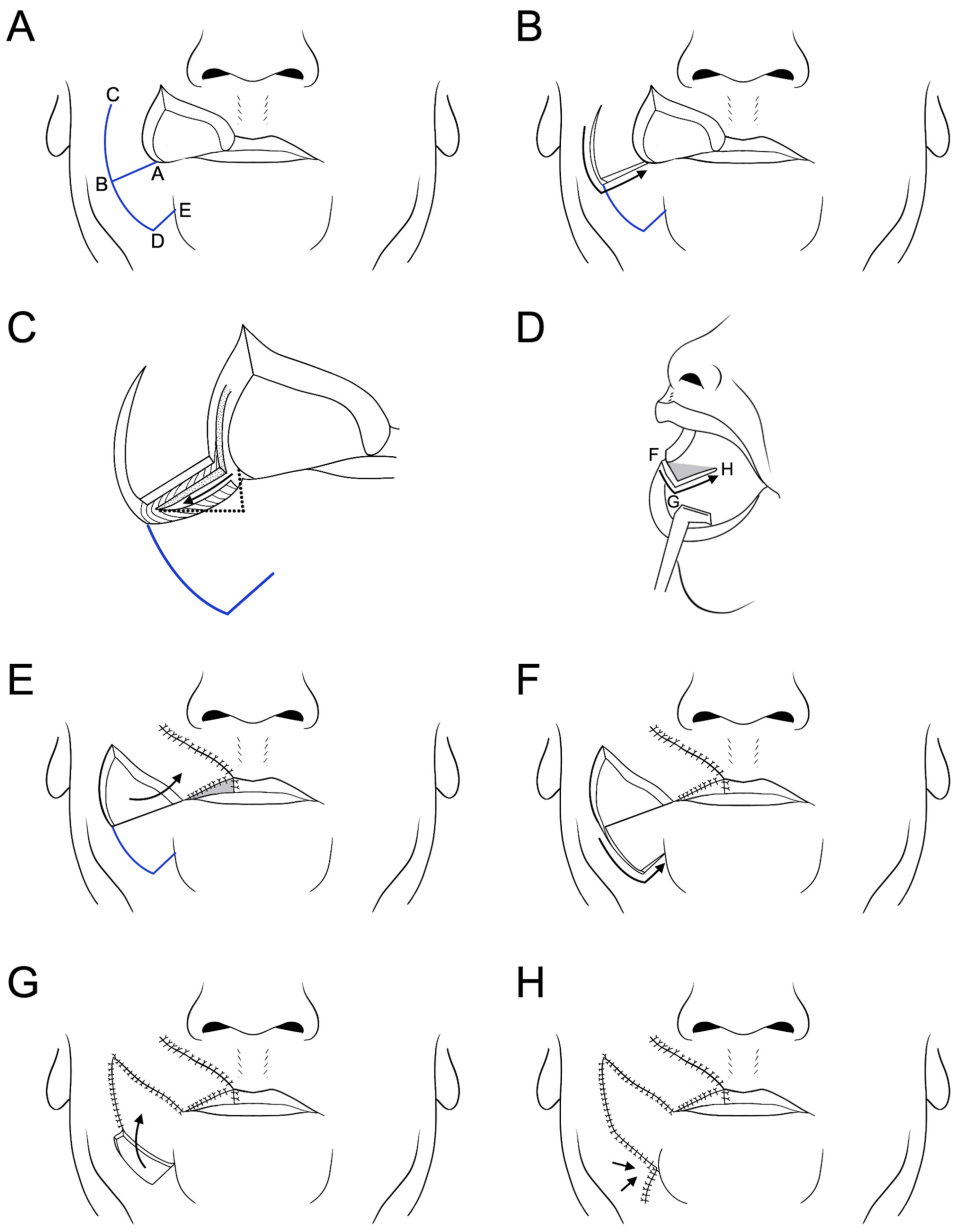
The surgical steps for the anchor flap. (A) The skin incision lines of an anchor flap. (B) After the skin incision to create the advancement flap. (C) The horizontal incision on the medial two-thirds was incised as full thickness except for the oral mucosa to create the mucosal flap (arrow). At this step, the medial two-thirds of the orbicularis oris muscle were incised, while the lateral one-third of the muscle was kept intact. The dotted line indicates the incision line for the new upper labial vermilion, which was created by means of an oral mucosal flap. (D) The incision line of the mucosa to create the mucosal flap. The gray area indicates the mucosal flap. (E) After rotating the advancement flap. The gray area indicated the new upper vermilion created by the mucosal flap. (F) After the skin incision to create the rotating flap. (G) After moving the rotating flap. (H) The remaining defect was sutured with primary closure. Image Credits: Figure 3 is an original image created by the author Masashi Kimura.

**Figure 4:**
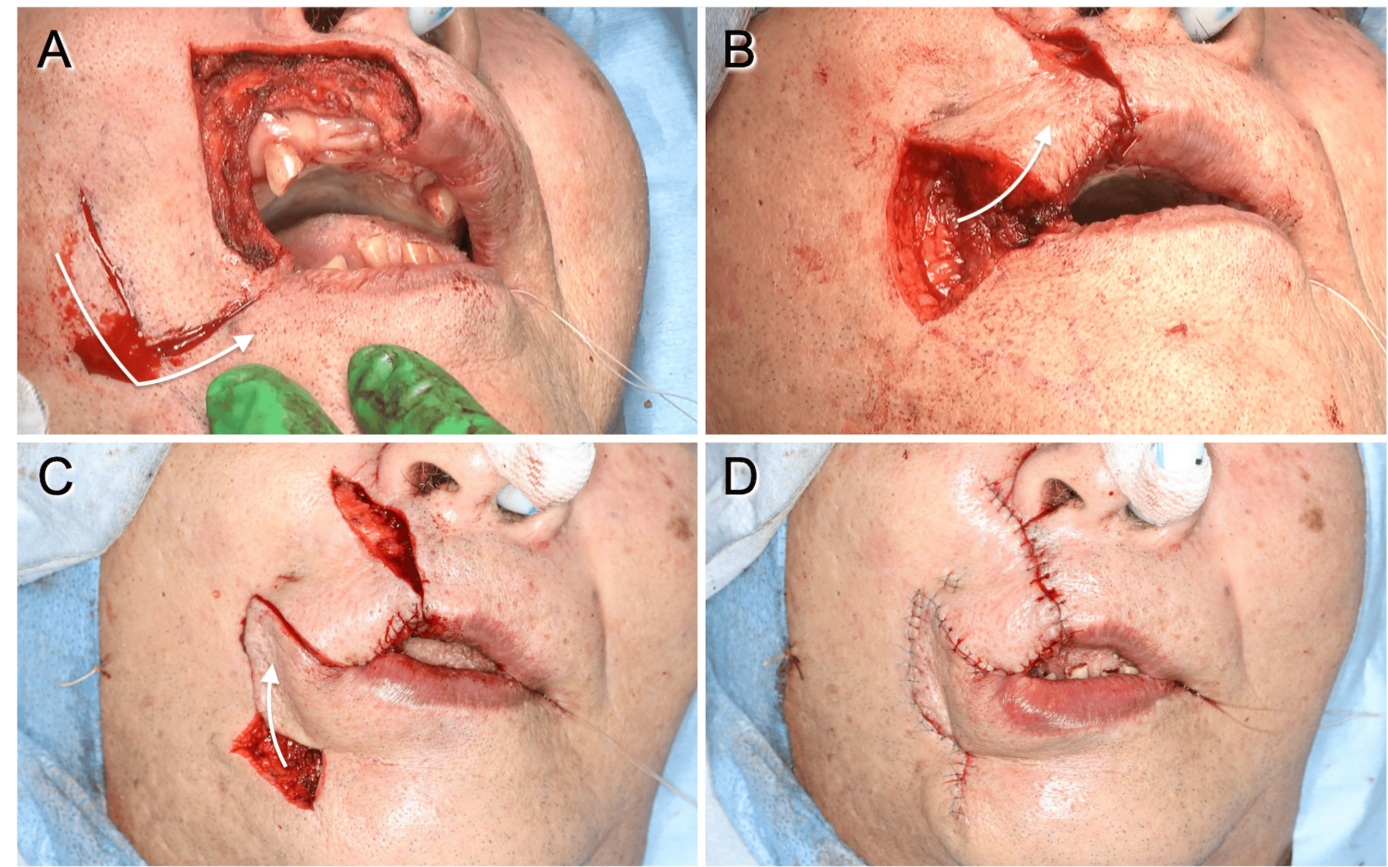
Intraoperative photographs. (A) The skin incision of the advancement flap (arrow). (B) After rotating the advancement flap (arrow). (C) After moving the rotating flap to cover the defect due to moving the advancement flap (arrow). (D) After being sutured with primary closure for the remaining defect.

Surgical resection with a 0.5 cm margin was planned according to the histologic diagnosis. The anticipated defect in the vermilion following resection measured approximately 3.5 cm, making primary closure unfeasible. Consequently, a modified reverse Yu's flap was planned with minor modifications (Figure 2A, 2B). The reversed Yu's flap consists of three components: an advancement flap of the upper lip (Figure 2A, A-B-C), a rotating flap from the buccal and mental regions (Figure 2A, B-D-E), and a mucosal flap created from the buccal mucosa to reconstruct the new vermilion (Figure 3D, F-G-H). Minor modifications were made to the advancement and rotating flaps to accommodate the larger defect. A horizontal incision to create the rotating flap running laterally from the corner of the mouth (Figure 2B, A-B) was set as 2.5 cm, slightly shorter than the defect to reduce the stress after flap rotation and suturing. Furthermore, the horizontal incision line was modified to fit the form of the vermilion by tilting it slightly downward (Figure 2B, A-B). For the rotating flap (Figure 2A, 2B, B-D-E), the original design positioned the vertical line (D-E) perpendicular to the horizontal incision (A-B) from the arcuate line and at a length equal to half the distance of D-A. The authors designed this vertical line to tilt slightly toward the midline to maintain the blood supply to the rotating flap (Figure 2B, D-E). This modified incision line, tailored to larger defects, was named the "anchor flap" because of the shape of its incision line (Figure 2B). All other procedures were performed as in previous reports [2,3,6,7]. The surgical steps for the anchor flap are shown in Figure 3. In brief, the horizontal incision on the medial two-thirds was made to full thickness, sparing the oral mucosa to create the mucosal flap (Figure 3B, 3C). The medial two-thirds of the orbicularis oris muscle at the commissure were incised, while the lateral one-third remained intact (Figure 3C). Advancement, rotating, and mucosal flaps were sequentially positioned to close the defect and sutured in place. These surgical steps are shown in Figure 3E-3H and Figure 4.

**Figure 5:**
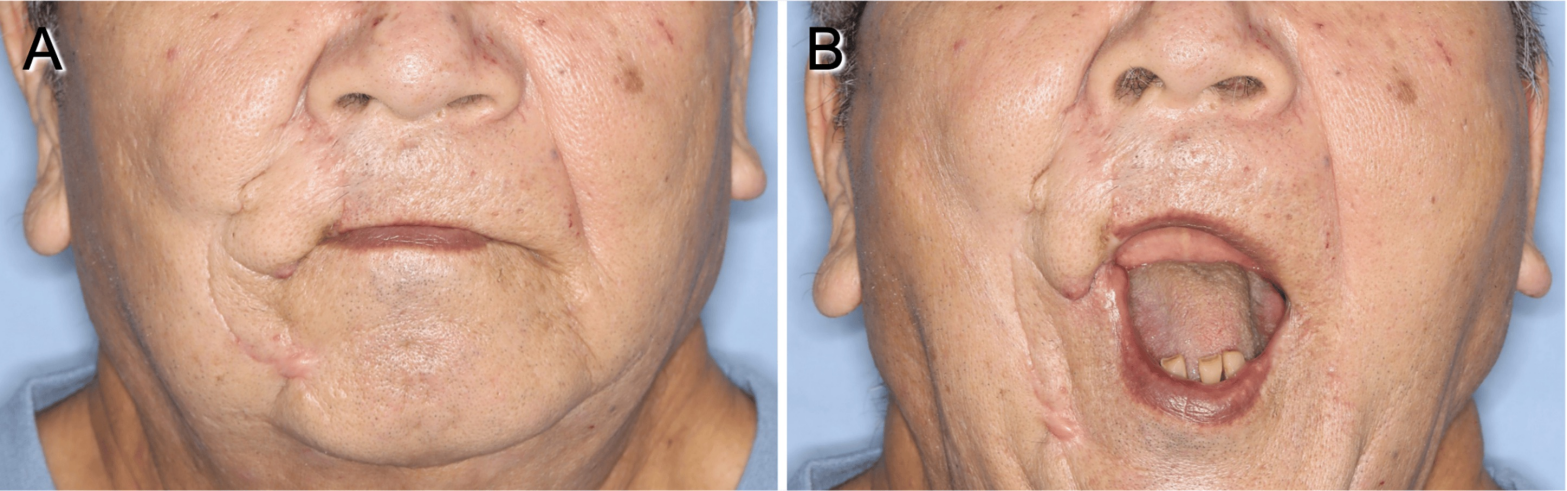
Postoperative views of the patient. (A) Postoperative view of the patient after nine months. Aesthetic results were satisfactory. (B) The patient had a functional outcome without trismus.

Postoperatively, minor marginal necrosis of the donor site in the rotating flap was observed and resolved with local wound care. Furthermore, poor wound healing with minor infection occurred in the medial portion of the advancement flap around the sutures, requiring debridement and secondary suturing two weeks post-surgery. However, no significant postoperative complications, such as total necrosis of the flaps or major wound dehiscence requiring secondary flap reconstruction, were observed. The patient achieved functional recovery without trismus, and the aesthetic results were satisfactory (Figure 5). The patient had a maximum mouth opening of more than 40 mm at two months postoperatively, and no further reduction in mouth opening was observed at one year postoperatively. There was no evidence of recurrence or metastases one year after surgery.

## Discussion

Reconstruction of lip defects is crucial as the lips play a pivotal role in maintaining aesthetic facial balance and physiological functions [2,8]. Lip defects can result from trauma, infectious diseases, congenital deformities, or, most commonly, tumor resection [2,9]. Primary closure is generally suitable for defects <1/3 of the lip, while larger defects usually require local flaps or free flaps. The Abbe, Estlander, and nasolabial flaps are the primary options for reconstructing upper lip defects affecting from one-third to two-thirds of the lateral upper lip [8].

The Abbe flap is a two-stage lip-switch flap based on the inferior labial artery, from which the tissue is harvested and transferred from the lower lip [5,8]. It allows preservation of the Cupid's bow, oral commissure, and modiolus position; however, it is an insensate flap and does not establish the continuity of the orbicularis oris. However, it is a two-stage procedure and complete flap loss is feasible. In addition, there is the possibility of microstomia resulting from the use of the lower lip as a donor site [10]. The Estlander flap is a lip-switch flap that is harvested and transferred from the lower lip, similar to the Abbe flap. Although this method is one-step, it also has the possibility to produce microstomia [8,11].

Nasolabial flaps are versatile and effective for repairing defects up to one-third of the upper lip, particularly when the vermilion is unaffected or in lateral defects with or without commissure involvement [8]. This flap is based on the facial artery and can achieve good aesthetic and functional outcomes. Superiorly based flaps are suitable for defects involving the central and lateral nasal dorsum, as well as the nasal tip and ala. An inferiorly based flap is useful for upper and lower lip defects [12]. However, nasolabial flaps do not restore the vermilion contour.

The Yu's flap, a single-stage sensate flap combining rotating and advancement flap, is primarily used for full-thickness lower lip defects [3]. The reverse Yu's flap is a modified method described in 2010 by Belmonte-Caro et al. and is a modified technique for upper lip defects [6]. Lateral defects up to half of the upper lip can be repaired with a unilateral reverse Yu's flap, while central defects or those affecting up to two-thirds of the upper lip require the bilateral reverse Yu's flaps [8]. The main advantage of this flap is its low risk of microstomia because it restores the vermilion form using the buccal mucosa as a mucosal flap [2]. The primary disadvantage of this method is that it is a relatively complicated procedure and may be more time-consuming than other local flaps [7]. 

## Conclusions

The reverse Yu's flap is useful for reconstructing defects of the upper lip up to one-half and has a low risk of microstomia because of the restoration of the vermilion using the buccal mucosa. In this report, we describe an anchor flap, which is a modified technique of the reverse Yu's flap, applied to a larger defect. An anchor flap was used to reconstruct a vermilion defect measuring approximately 3.5 cm, and the patient had a functional outcome without trismus. An anchor flap can be a valuable method for larger defects of the upper lip and can achieve good aesthetic and functional results.

## References

[1] Lee J, Oh SJ, Jung SW, Koh SH (2012). Combined rotation and advancement flap reconstruction for a defect of the upper lip: 2 cases. Arch Plast Surg.

[2] Li ZN, Li RW, Tan XX (2013). Yu's flap for lower lip and reverse Yu's flap for upper lip reconstruction: 20 years experience. Br J Oral Maxillofac Surg.

[3] Yu J (1989). A new method for reconstruction of the lower lip after tumor resection. Eur J Plast Surg.

[4] Daya M, Nair V (2009). Free radial forearm flap lip reconstruction: a clinical series and case reports of technical refinements. Ann Plast Surg.

[5] Abbe R (1968). A new plastic operation for the relief of deformity due to double harelip. Plast Reconstr Surg.

[6] Belmonte-Caro R, Infante-Cossio P, Garcia-Perla-Garcia A, Torres-Carranza E (2010). Reverse Yús flap for upper lip reconstruction. J Plast Reconstr Aesthet Surg.

[7] Sanchez-Sanchez M, Infante-Cossio P, Lozano-Rosado R (2017). Resection of upper lip adenoid cystic carcinoma and reconstruction with reverse Yu flap: report of three cases and a literature review. Mol Clin Oncol.

[8] Boson AL, Boukovalas S, Hays JP, Hammel JA, Cole EL, Wagner RF Jr (2021). Upper lip anatomy, mechanics of local flaps, and considerations for reconstruction. Cutis.

[9] Akbas H, Karacaoglan N (2001). Reconstruction of large lower lip defects: a new method. Otolaryngol Head Neck Surg.

[10] Sowa Y, Inafuku N, Kodama T, Morita D, Numajiri T (2019). Medial upper lip vermillion reconstruction with a labial artery-based cross-lip vermillion flap. Plast Reconstr Surg Glob Open.

[11] Kumar A, Shetty PM, Bhambar RS, Gattumeedhi SR, Kumar RM, Kumar H (2014). Versatility of Abbe-Estlander flap in lip reconstruction - a prospective clinical study. J Clin Diagn Res.

[12] Bayer J, Schwarzmannová K, Dušková M, Novotná K, Kníže J, Sukop A (2018). The nasolabial flap: the most versatile method in facial reconstruction. Acta Chir Plast.

